# Laryngeal Spasm in Children

**Published:** 1907-08-17

**Authors:** 


### Laryngology and Rhinology.

### LARYNGEAL SPASM IN CHILDREN.

Children are much more liable than adults to spasm of the glottis as a result of local or reflex irritation and, by reason of the small size of the larynx, a degree of narrowing which in the adult would produce no symptoms causes in a child severe and urgent dyspnœa. Spasm plays an important part in all forms of laryngeal disease in children, and therefore their dyspnœa is characterised by exacerbations, which nearly always occur at night, and by intermissions either complete or partial, according as the spasm is the sole or the partial cause of the dyspnœa. The symptoms of glottic spasm are much the same whatever the cause. In the case, for instance, of an ordinary catarrhal laryngitis, with slight pyrexia and hoarseness, the child may be suddenly awakened in the middle of the night by a fit of coughing; spasm of the glottis ensues with inspiratory stridor, retraction of the epigastrium and lower ribs, cyanosis, and all the signs of asphyxia, which persist until death appears imminent; the spasm then suddenly relaxes, air enters with a prolonged crowing sound, the colour returns, and the child usually soon goes to sleep. The attack may or may not recur the same night, but usually returns for the next few nights, though with diminishing severity; in the day-time there is no spasm, but the child has a cold and is hoarse.

### Laryngitis Stridula.

This is a case of catarrhal laryngitis complicated, as it often is, with spasm; it is called spasmodic laryngitis or laryngitis stridula. Although rarely fatal, it is a condition which gives rise to much anxiety; the attacks are terrifying to the friends, and for the physician there may be much difficulty in excluding more serious disease. In cases of œdematous laryngitis the obstruction is due to swelling at the upper aperture of the larynx or in the subglottic region; the temperature is higher and the child appears more ill than in spasmodic laryngitis. The dyspnœa rapidly increases and becomes continuous, for in children œdema of the larynx may come on with great rapidity. It is usually possible, even in very young children, to get a glimpse of the swollen arytenoids with the laryngeal mirror, but, if the attempt disturbs the child greatly, it is better to desist and to palpate the part quickly with the finger.

### Laryngeal Diphtheria.

The symptoms at the onset of laryngeal diphtheria are similar to those of spasmodic laryngitis, for the dyspnœa is at first largely due to spasm, and is subject to exacerbations; as the membrane grows this symptom becomes continuous and more severe. The temperature is not very high, rarely above 103° F., nor is the child very ill at first; there is nearly always membrane on the fauces, and in the rare primary laryngeal cases the presence of albumen in the urine and enlargement of the cervical glands will aid the diagnosis. A foreign body in the larynx causes violent laryngeal dyspnœa; usually it is known that some body has been inhaled from the mouth, but sometimes no history can be obtained. A foreign body may be suspected if a very urgent attack of dyspnœa occur suddenly in a child previously in perfect health. The first attack may prove fatal, but, if the body be small, the spasm generally subsides to recur in exacerbations with varying degrees of hoarseness and dyspnœa in the intervals. Laryngeal papillomata are not uncommon in children, and cause recurrent attacks of spasm; it may be said that chronic long-continued hoarseness in a child, without signs of catarrh or any constitutional disease, is usually due to papilloma.

### Laryngismus Stridulus.

An important form of spasm occurs in children who have no local disease in the larynx. This is laryngismus stridulus, a purely spasmodic affection which occurs in unhealthy, ill-nourished children between six months and two years of age, and usually in association with rickets; the predisposing condition is increased excitability of the nervous system, and the exciting cause of the reflex comes from the alimentary tract (gastritis, worms, etc.), or from the pharynx and especially from the presence of adenoids. The attacks may recur several times in a day; they begin with a few short, noisy inspirations, followed by cessation of breathing, and end with a long crowing inspiration, and they are sometimes fatal; nurses speak of them as a "passion fit" or "holding the breath." This affection differs from all those described above, in that there is nothing abnormal—no cough or hoarseness—in the intervals between the attacks.

On account of the risk of spasm and œdema, catarrhal laryngitis in children should always receive careful attention; should spasm occur, an emetic dose of ipecacuanha generally gives rapid relief, or an inhalation of amyl nitrite may be given; a few whiffs of choloroform will check the spasm, but the physician can seldom arrive in time to administer it. Small doses of bromides may be recommended between the attacks, or nitroglycerine in doses of $\frac{1}{500}$ grain. In the treatment of laryngismus stridulus tonics and change of air to a higher locality are of great importance; overeating must be avoided, and free action of the bowels maintained. The measures mentioned above may be used during the spasm, or the child may be put into a warm bath and cold water poured on to the head. The quickest way of relieving the spasm is to hook forward the epiglottis with the finger, and the mother may be taught to do this; stimulation of the nasal mucous membrane or of the conjunctiva sometimes has a good effect. Tracheotomy and intubation are rarely needed; nor indeed is there time to perform such operations in a bad case.

